# Successful endoscopic retrograde appendicitis therapy for acute appendicitis using a multi-side-hole conical cap

**DOI:** 10.1055/a-2718-4790

**Published:** 2025-11-06

**Authors:** Lu Qiao, Longbao Yang, Juhui Zhao, Cui Fu, Shangxuan Cai, Shiyang Ma

**Affiliations:** 1 117799 Department of Gastroenterology, Second Affiliated Hospital of Xi’an Jiaotong University, Xi'an, China

Endoscopic retrograde appendicitis therapy (ERAT), a novel minimally invasive technique, has gained attention for treating acute simple appendicitis 1 . However, cannulation in ERAT is challenging, particularly in cases of appendiceal orifice stenosis secondary to acute inflammation 2 . This case report describes our innovation of a multi-side-hole conical cap to address this challenge. A 53-year-old woman presented with right lower quadrant abdominal pain for 1 day. Physical examination revealed significant tenderness in the right lower quadrant. Blood tests showed leukocytosis (13 × 10 ^9^ /L), and an abdominal ultrasound indicated appendicitis. After emergency admission and bowel preparation, ERAT was performed. During the colonoscopy, a large amount of fecal residue can be seen remaining in the intestinal cavity. Using the multi-side-hole conical cap ( Fig. 1 **a** ), we successfully cleared the fecal residues entered the appendiceal cavity ( Fig. 1 **b** ). The cholangioscopy showed white pus in the appendiceal cavity, which was irrigated with metronidazole-saline solution ( Fig. 1 **c** ). Finally, a 7 Fr × 5 cm pancreatic stent (Cook, Ireland, SPSOF-7-5) was deployed ( Fig. 1 **d** , Video 1 ). Within 24 hours postoperatively, the patient’s abdominal pain significantly subsided and was discharged. At the 2-month follow-up, the patient reported no specific discomfort. Emergency bowel preparation might be inadequate with more fecal residues. In this situation, we innovated a multi-side-hole conical cap to aspirate and flush away these fecal residues. This cap, based on the original conical cap, features two rows of circular holes to enhance suction during cholangioscopy (Micro-Tech, China, CDS22001) insertion. Compared to standard conical caps, it removes most fecal residues and flushes out those trapped within the cap, facilitating smoother entry into the cecum and appendiceal cavity. In conclusion, the multi-side-hole conical cap is a promising tool for improving the success rate of ERAT in acute appendicitis patients, especially in patients with poor intestinal preparation. Further studies are needed to validate its efficacy and safety.

**Fig. 1:**
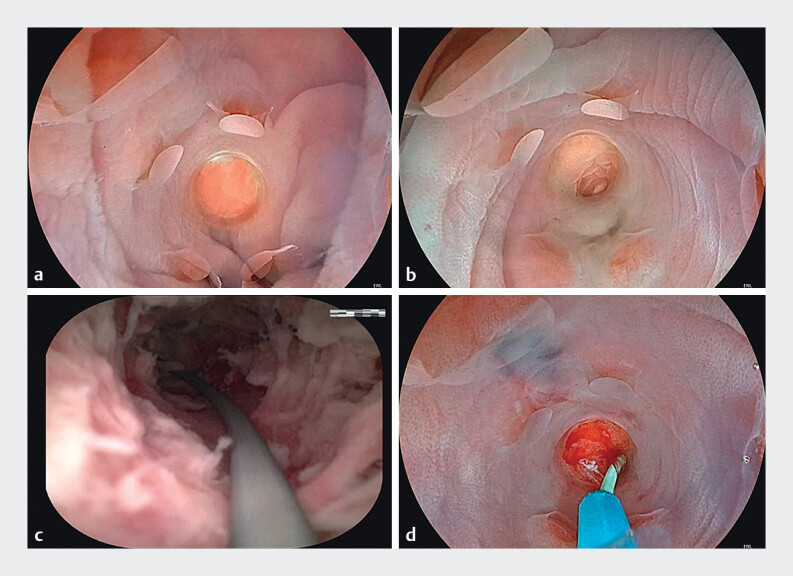
Process of ERAT with multi-side-hole conical cap. **a** A multi-side-hole conical cap was installed on the front end of the colonoscope. **b** The colonoscope was successfully passed to the appendiceal orifice. **c** A pus-filled infection was seen inside the appendiceal lumen and a guidewire was placed. **d** A stent was placed under guidewire guidance.

Using a multi-side-hole conical cap during ERAT facilitates easier removal of fecal debris, resulting in a clearer visual field-especially in patients with suboptimal bowel preparation.Video 1

Endoscopy_UCTN_Code_TTT_1AQ_2AJ

## Notes

Endoscopy E-Videos https://eref.thieme.de/e-videos
*E-Videos* is an open access online section of the journal *Endoscopy* , reporting on interesting cases and new techniques in gastroenterological endoscopy. All papers include a high-quality video and are published with a Creative Commons CC-BY license. Endoscopy E-Videos qualify for HINARI discounts and waivers and eligibility is automatically checked during the submission process. We grant 100% waivers to articles whose corresponding authors are based in Group A countries and 50% waivers to those who are based in Group B countries as classified by Research4Life (see: https://www.research4life.org/access/eligibility/ ). This section has its own submission website at https://mc.manuscriptcentral.com/e-videos .
